# Preparation and Characterization of Extruded Composites Based on Polypropylene and Chitosan Compatibilized with Polypropylene-Graft-Maleic Anhydride

**DOI:** 10.3390/ma10020105

**Published:** 2017-01-25

**Authors:** Fernando Javier Carrasco-Guigón, Dora Evelia Rodríguez-Félix, María Mónica Castillo-Ortega, Hisila C. Santacruz-Ortega, Silvia E. Burruel-Ibarra, Jose Carmelo Encinas-Encinas, Maribel Plascencia-Jatomea, Pedro Jesus Herrera-Franco, Tomas Jesus Madera-Santana

**Affiliations:** 1 Departamento de Investigación en Polímeros y Materiales, Universidad de Sonora, Hermosillo 83000, Sonora, Mexico; fernandocarrascog84@gmail.com (F.J.C.-G.); monicac@guaymas.uson.mx (M.M.C.-O.); hisila@polimeros.uson.mx (H.C.S.-O.); silvia@gimmunison.com (S.E.B.-I.); carmelo@polimeros.uson.mx (J.C.E.-E.); 2 Departamento de Investigación y Posgrado en Alimentos, Universidad de Sonora, Hermosillo 83000, Sonora, Mexico; mplascencia@guayacan.uson.mx; 3 Unidad de Materiales, Centro de Investigación Científica de Yucatán, Mérida 97200, Yucatán, Mexico; pherrera@cicy.mx; 4 Laboratorio de Envases, CTAOV, Centro de Investigación en Alimentos y Desarrollo A.C, Hermosillo 83304, Sonora, Mexico; madera@ciad.mx

**Keywords:** polypropylene, chitosan, composites, maleic anhydride, extrusion

## Abstract

The preparation of composites of synthetic and natural polymers represent an interesting option to combine properties; in this manner, polypropylene and chitosan extruded films using a different proportion of components and polypropylene-graft-maleic anhydride (PPgMA) as compatibilizer were prepared. The effect of the content of the biopolymer in the polypropylene (PP) matrix, the addition of compatibilizer, and the particle size on the properties of the composites was analyzed using characterization by fourier transform-infrared spectroscopy (FT-IR), scanning electron microscopy (SEM), differential scanning calorimetry (DSC), tensile strength, and contact angle, finding that in general, the addition of the compatibilizer and reducing the particle size of the chitosan, favored the physicochemical and morphological properties of the films.

## 1. Introduction

Polypropylene (PP) is the most important material among polyolefins used as a matrix in polymer composites because of its relatively superior properties such as high melting temperature (Tm), excellent mechanical and thermal properties, and low density [1,2]. However, the large production and use of synthetic polymers lead to the accumulation of waste plastic products, creating a serious source of pollution that affects the environment [3,4,5]. The replacement of synthetic polymers by biodegradable natural polymers would be the perfect solution to this problem; however, natural polymers do not possess the excellent physicochemical properties of synthetic polymers or their easy processing.

The preparation of mixtures of synthetic and natural polymers represent a simple way to combine their best properties, obtaining materials with acceptable morphological, thermal, and mechanical properties, and shorter degradation time compared to synthetic polymers. Therefore, the partial biodegradation of these polymeric blends is one alternative that could contribute to minimizing environmental pollution [6,7].

Chitosan is a partially deacetylated polymer of acetyl glucosamine obtained by alkaline deacetylation of chitin, the second most abundant natural polysaccharide, after cellulose. Chitosan has interesting properties such as biocompatibility, biodegradability, non-toxicity, and antimicrobial activity; therefore, its films have a great potential to be used as packaging materials [8,9,10,11,12].

The PP matrices are of a hydrophobic nature; therefore, they repel the polar sites of chitosan. The resulting blend of these two types of polymers is generally immiscible. Polymers grafted with maleic anhydride have been used as compatibilizers for immiscible binary mixtures, showing good results [13,14,15,16].

In our previous work, we reported the preparation and characterization of extruded polyethylene films and chitosan, using the extrusion molding method, which is widely used in industry [4]. Another polymer of great use at the industrial level is polypropylene; we consider the study of polypropylene for this type of polymer system to be very interesting. Therefore, the objective of the present work is to obtain extruded composites of polypropylene and chitosan.

In this paper, we report the preparation of polypropylene and chitosan extruded films using different formulations of the components; as well as, the effect of the addition of polypropylene-graft-maleic anhydride (as a compatibilizer) in the polymer blends. These composites were characterized by infrared spectroscopy, scanning electron microscopy, tensile strength, contact angle, and by their thermal properties.

## 2. Results and Discussion

### 2.1. Extruded Composites of Polypropylene and Chitosan

Extruded composites of PP and chitosan using glycerol as plasticizer were obtained. It was possible to prepare films with a 9% (w/w) maximum content of chitosan and with dimensions of 1.5 cm width and 0.4 mm thickness. Because polypropylene is a hydrophobic material and chitosan is hydrophilic, the combination of both polymers results in the production of a nonhomogeneous material; to improve the miscibility of polymers of different nature, it is recommended to use a maleic anhydride based compatibilizer, such as maleic anhydride grafted polypropylene (PPgMA), in a proportion of 5% (w/w) [4,12].

### 2.2. Extruded Composites of Polypropylene and Chitosan with Compatibilizer

Extruded composites of PP and chitosan using PPgMA and glycerol as compatibilizer and plasticizer, respectively, were obtained. It was possible to prepare films with a 9% (w/w) maximum content of chitosan and with dimensions of 1.5 cm width and 0.4 mm thickness. It was not possible to obtain films with a higher content of chitosan because the resulting films had too many holes, were very fragile, and were difficult to process using this method of extrusion. However, this chitosan content in the composites is an acceptable value for this processing method, considering that chitosan is difficult to extrude to obtain thin films because the chitosan degrades before melting; for this purpose, the solvent evaporation method (casting) is a suitable option, however extrusion is the most economical option [17]. In the literature, there have not been reports published regarding extruded PP and chitosan composites, so the method of preparation of these composites is novel and very useful industrially. Several authors have published composites based on chitosan and PP using a high content of chitosan; however, these materials are obtained by methods such as casting or thermocompression [18,19]. On the other hand, the preparation of extruded chitosan/polyester blends has been reported in a twin screw extruder utilizing up to 70% (w/w) of chitosan, but was subsequently processed by injection molding to obtain bars [20]. Another interesting study reported the preparation of films of previously plasticized chitosan and polyethylene using a single screw extruder; in this study, it was possible to obtain films with up to 10% (w/w) of chitosan [21]. Previously, we reported the production of extruded films of chitosan/polyethylene with acceptable properties that had 20% (w/w) of chitosan [4]. However, using PP as a matrix, it was not possible to achieve that chitosan content in the extruded film because of difficulty during processing.

### 2.3. FT-IR Spectroscopy Analysis

**Figure 1:**
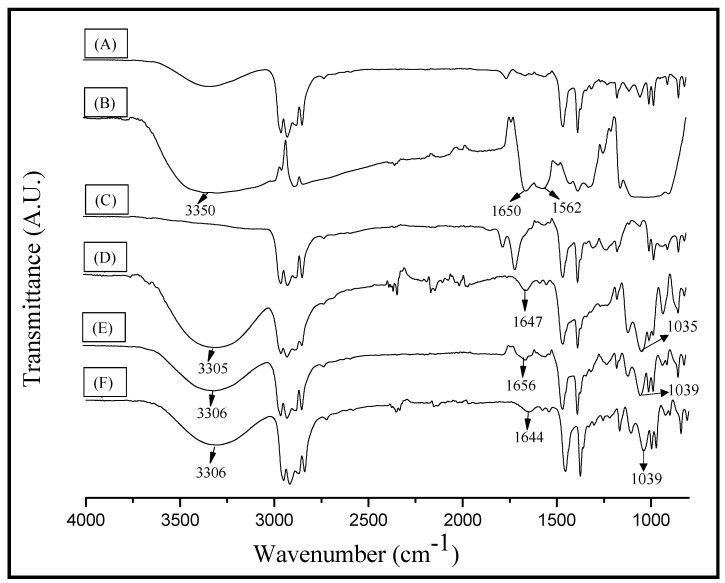
Infrared spectroscopy of (**A**) PP; (**B**) Chitosan; (**C**) PPgMA; (**D**) A9; (**E**) B9; (**F**) C9.

**Figure 2:**
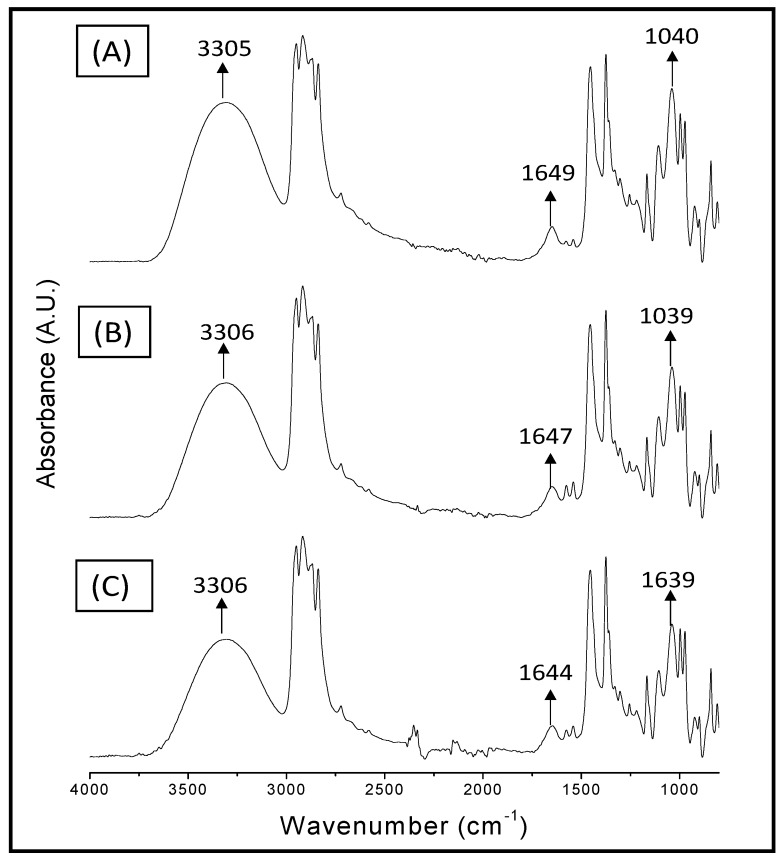
Infrared spectroscopy of (**A**) C5; (**B**) C7; (**C**) C9.

The infrared spectrums showed the characteristic signals of the individual polymers (Figure 1); the IR spectrum of PP (Figure 1A) shows: a stretching vibration of -C-H at a wavenumber around 2800–3000 cm^−1^, a bending vibration of -CH_2_ at 1453 cm^−1^, and a bending vibration of -CH_3_ at 1375 cm^−1^ [22]; the PPgMA spectrum (Figure 1B) shows signals characteristic of PP and additionally the signal corresponding to the carbonyl group (1709 cm^−1^) of maleic anhydride. The main signals for chitosan appear at 3352 cm^−1^ (O-H stretch), 2879 cm^−1^ (C-H stretching), 1650 cm^−1^ (C=O stretching, amide I), 1563 cm^−1^ (N-H bending vibrations), 1415 cm^−1^ (C-N stretching), and 1039 cm^−1^ (C-O-C signal) [2,23]. The spectra of the A9, B9, and C9 films (Figure 1D,E) present the spectral contributions of the PP, and chitosan; B9 and C9 also present the spectral contributions of PPgMA. The signal at 3352 cm^−1^ corresponding to the O-H stretching of chitosan shifted to a lower wavenumber (around 3306 cm^−1^) in the films of PP and chitosan with or without compatibilizer; this displacement can be attributed to the presence of glycerol as a plasticizer in the films. Moreover, the signal at 1650 cm^−1^ corresponding to amide I of chitosan showed a slight displacement of 6 cm^−1^ for the B9 and C9 films, indicating the possible existence of interaction between the -NH_2_ group of chitosan and the polar group of the compatibilizer. On the other hand, the film A9 showed the C-O-C signal of chitosan with the highest intensity compared to B9 and C9, which can be attributed to the heterogeneity of the A9 film because it does not contain compatibilizer, so that a larger chitosan agglomerate could be involved during the analysis. Figure 2 shows that the C7 film also exhibited shifts in the chitosan signals corresponding to the OH group and amide I; while the C5 film showed no displacements in this signal, which is attributed to the lower amount of chitosan in the composite; therefore, the intermolecular interactions of this biopolymer cannot be appreciated during the analysis. However, an increase in the intensity of the signal corresponding to the OH group and the C-O-C bond of chitosan in relation to the content of chitosan in the film was not observed, as expected; probably due to the small difference in the chitosan content in the film and the non-homogeneous dispersion of this biopolymer in the PP matrix.

### 2.4. Scanning Electronic Microscopy

**Figure 3:**
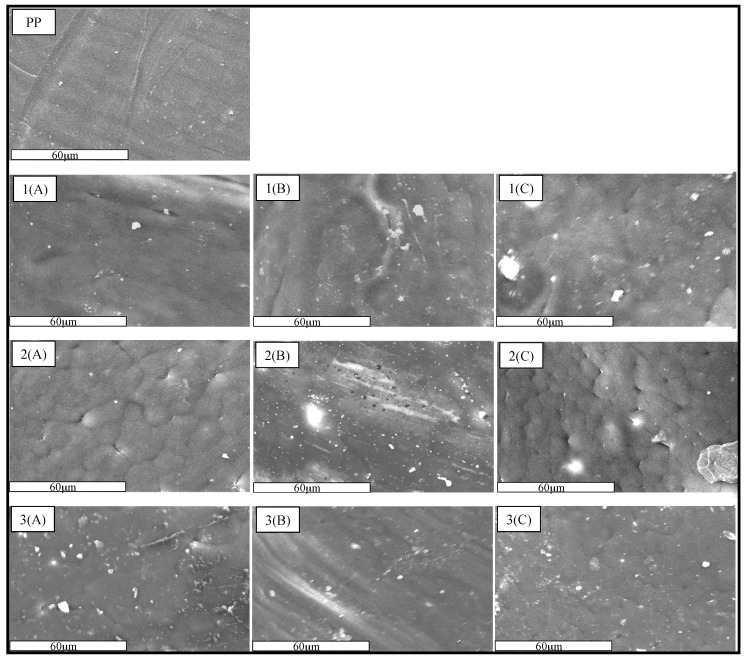
SEM Images: (**A**–**C**) films with 5%, 7%, and 9% of chitosan content, respectively; (1) films without compatibilizer; (2) films with compatibilizer; (3) films with milled chitosan and compatibilizer. 1000× magnification.

The studies by scanning electron microscopy (Figure 3) showed that the surface of the PP film is homogeneous and the addition of the biopolymer affected this characteristic due to the formation of agglomerates in the polymer matrix. It was also observed that the increase of chitosan content in the films affected their homogeneity (Figure 3(1A–1C)), because of the difficulty to obtain a homogeneous dispersion of chitosan by the extrusion processing method. Additionally, its hydrophilic character does not allow its homogeneous dispersion in the PP matrix that is hydrophobic; however, the addition of compatibilizer improved the homogeneity of the films, as shown in Figure 3(2A–2C). This behavior is consistent with the literature where it was reported that the use of polymers grafted with maleic anhydride are good compatibilizers for immiscible binary mixtures [13,14,15,16]. Moreover, films with smaller chitosan particle size (Figure 3(3A–3C)) showed better dispersion of the particles of chitosan in the PP matrix, so in these films the formation of agglomerates is almost unnoticeable, which is attributed to the good dispersion of the chitosan particles in the PP matrix. Based on these results, we can deduce that the homogeneity of the films was promoted by the addition of the compatibilizer and by the reduction of the chitosan particle size.

### 2.5. Contact Angle

The values of the contact angle of the PP film and PP/chitosan composites are shown in Table 1, where it can be seen that the polypropylene film showed a hydrophobic behavior presenting a contact angle of 104°, while the PP/chitosan films showed values between 82.7° and 74.5°; this decrease in the contact angle is attributed to the addition of chitosan in the PP matrix, attributed to the hydrophilic character of this biopolymer. In general, the contact angle slightly decreases with increasing chitosan content in the film; except for the film with compatibilizer, where a change in the contact angle values related to the increase of chitosan content is not observed. This behavior can be explained considering the hydrophobic character of the PPgMA. In chitosan films with smaller particle sizes, the effect of the addition of a compatibilizer is not observed, because the good dispersion of chitosan particles in the PP matrix (observed by SEM analysis) promotes hydrophilicity of the composite [24,25]. The addition of chitosan of hydrophilic nature in the hydrophobic matrix of PP increases the hydrophilic character of the composite as well as the roughness of the surface due to the formation of chitosan agglomerates, as observed by SEM analysis; both situations can favor the decrease of the contact angle, according to the reports in the literature where it is mentioned that in systems with good wettability, the increase of the roughness leads to smaller contact angles.

### 2.6. Thermal Properties

Table 2 shows the thermal properties obtained by differential scanning calorimetry in films of PP with different contents of chitosan. PPgMA had a lower melting point and degree of crystallinity than pure PP, therefore the insertion of maleic anhydride into the PP structure affects the packaging of the polymer chains. The addition of chitosan in the PP matrix did not reveal any important effect on the melting point (T_m_) of PP, and it can be deduced that the PP matrix and the chitosan phase are only partially miscible. The crystallinity of PP decreased with the presence of chitosan [2], which indicates that the chitosan particles settled between the polypropylene chains, hindering the ordering of the PP chains. In addition, the films with higher content of chitosan (A9, B9, and C9) had the lowest crystallinity index values, because the hindering of the alignment of the PP chains increases with the content of chitosan in the film. Moreover, the films without compatibilizer had a slightly higher index of crystallinity compared to the films with PPgMA, indicating that it showed a slight hindrance on the order of the synthetic polymer chains. Reducing the particle size of the chitosan favored crystallinity of the composite, shown in the C5 film, however with increasing content of chitosan (film C9) this behavior was not observed, which could explain the increased chitosan content in the PP matrix that caused the formation of agglomerates, which resembles the situation of using chitosan with larger particle sizes. Mucha and Królikowski reported that an organic filler such as chitosan forms an amorphous inclusion in the composites on which isotactic PP cannot be adsorbed [26].

### 2.7. Mechanical Properties

Table 3 presents Young´s modulus, tensile strength, and elongation at break properties of PP/chitosan composites. In general, the mechanical properties of the PP film decrease with the addition of chitosan. The modulus of elasticity of the PP film was 1484 MPa. It was observed that the addition of chitosan to the PP matrix decreased stiffness of the film and this behavior can be attributed to the particles of chitosan affecting the ordering of the PP chains, thereby changing the crystallinity of PP; this was demonstrated by the DSC analysis and this affects the stiffness of the material. It was also observed that in general, the increase in the chitosan content increases the Young’s modulus of the composite; which can be explained based on the rigidity of chitosan. Moreover, the films without compatibilizer showed the lowest values of the Young’s modulus, while films with chitosan of smaller particle size and compatibilizer showed the highest values of the Young’s modulus, allowing us to deduce that the uniform distribution of the chitosan particles in the PP matrix has an important influence on this property of the films. The behavior of tensile strength with an increase in the content of chitosan is not clear; however, just as in the previous case, the films without compatibilizer showed the poorest properties, while homogeneous films, according to the SEM studies (C5–C9), showed the highest values of tensile strength. Finally, the increase of chitosan content did not cause an apparent change of the elongation at break in the A5–A9 and B5–B9 films, while an increase of this property in the C5–C9 films was observed, which was attributed to the few zones of imperfection of these films. This was because the chitosan particles are distributed uniformly in the PP matrix, which promotes the interaction of these polymers, as well as the interfacial adhesion, as revealed by the FT-IR, SEM, and DSC studies. In general, the mechanical properties of the PP matrix decrease with the addition of chitosan; so we deduce that the biopolymer acts as a filler in the composite. In our previous papers, based on PE and chitosan composites, the mechanical properties also decreased with the exception of the Young’s modulus, which increased with the addition of chitosan because this biopolymer is more rigid than PE [4,12]. Moreover, when flax fibers are added to the PP matrix the mechanical properties are favored, which demonstrated that the biopolymer is acting as a reinforcement for the matrix [26].

In general, the mechanical properties and crystallinity of the PP were affected by the addition of chitosan in PP/chitosan composites; therefore, we deduce that the biopolymer acts as a filler in the composite. In contrast, when flax fibers are added to the PP matrix, these properties are favored, which shows that in this case the biopolymer is acting as a reinforcement and at the same time as a nucleating agent, respectively [27]. In relation to the morphology of this type of extruded composites, it is difficult to obtain a homogeneous dispersion of the biopolymer in the polymer matrix, as this characteristic is favored when the material is prepared using the method of evaporation of solvents; however, this is more expensive and less environmental friendly. It is necessary to obtain materials with acceptable, low-cost, and environmentally friendly properties. It is important to emphasize that the use of chitosan in this type of composite adds potential antimicrobial activity that extends the potential applications of the material under study.

## 3. Materials and Methods

### 3.1. Materials

Chitosan of medium molecular weight (190,000–310,000 Da, and 75%–85% deacetylated) and Polypropylene-graft-maleic anhydride (PPgMA, with 8%–10% (w/w) maleic anhydride) were obtained from Sigma-Aldrich (St. Louis, MO, USA). Glycerol was obtained from J.T. Baker (Corporate Parkway, Center Valley, PA, USA). Highly isotactic polypropylene (PP) and Formolene 4100 (density of 0.9 g/cm^3^) was obtained from the Formosa Company (Livingston, NJ, USA). PP and PPgMA were milled; chitosan was milled and sieved and was dried at 110 °C for 24 h before usage, and glycerol was used as received.

### 3.2. Preparation of the Composites

The films of polypropylene/chitosan were prepared using PPgMA as a compatibilizer and glycerol as a plasticizer for chitosan because of its plasticizing power, nontoxicity, and thermal stability [4,28].

**Figure 4:**
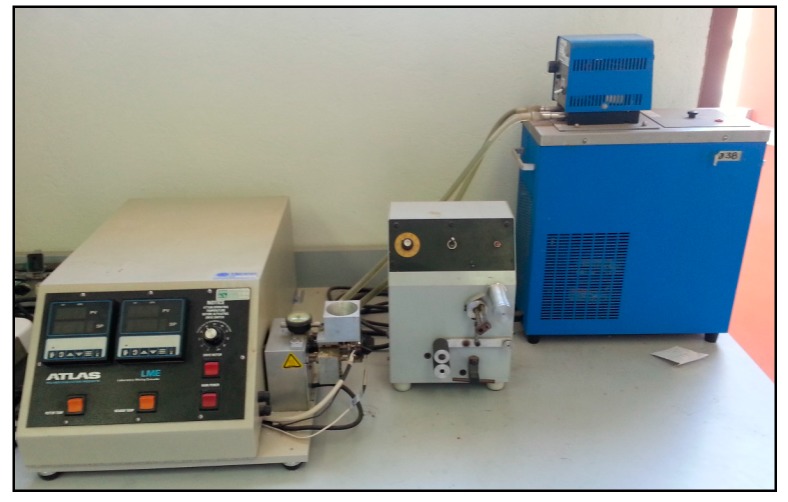
Image of the extruder used in the preparation of the films.

The polymer composites were prepared by mechanically mixing chitosan and glycerol for 10 min, and then PP and PPgMA were added and continuously mixed for 10 min until a homogeneous mass was obtained. This mixture was added to an Atlas laboratory mixer-extruder (Plantation, FL, USA) with a single screw (Figure 4); using a speed of 40 rpm. The temperatures were controlled at 175 °C and 185 °C for the rotor and the head, respectively. In order to study the effect of particle size of the chitosan on the physicochemical properties of the extruded films, a reduction of the particle size of this biopolymer using a Tyler mesh #100 (yielding an average particle size of 150 µm) was carried out. The formulations of the PP/chitosan composites are provided in Table 4. The content of compatibilizer in the films was 5% (w/w) as recommended in our previous publications [4,12].

### 3.3. Characterization

#### 3.3.1. FT-IR Spectroscopy

FT-IR spectroscopy analyses were performed using a Thermo Fisher Scientific spectrophotometer, Nicolet iS5 model (Thermo Fisher Scientific, Waltham, MA, USA). The spectrum scan was carried out from 4000 to 400 cm^−1^. The measurement was realized by an ATR accessory in the mode of transmittance and absorbance. Square samples of 1 cm^2^ area were used.

#### 3.3.2. Scanning Electronic Microscopy

The morphology of the films was examined using a JEOL 5410LV Scanning Electron Microscope (SEM) (JEOL, Tokyo, Japan), equipped with an INCA dispersive X-ray detector system (Oxford Instruments, Austin, TX, USA), and operated at a voltage of 20 kV. The samples were adhered to the sample holder with carbon tape and were gold-sputtered before the SEM examination.

#### 3.3.3. Contact Angle Determination

The contact angle with a water droplet was measured to determine the hydrophilic-hydrophobic behavior of the composites; this study was carried out with a Dataphysics Instruments GmbH model OCA15SEC (Dataphysics Instruments GmbH, Filderstadt, Germany).

#### 3.3.4. Thermal Analysis

The thermal behavior was studied by differential scanning calorimetry (DSC) using a Perkin Elmer model 8500 (Perkin Elmer, Waltham, MA, USA); approximately 6 mg of sample were placed in the aluminum pan at a heating rate of 10 °C·min^−1^ from −40 to 250 °C, under nitrogen atmosphere. The melting temperature and enthalpy of the films were automatically calculated by the software provided with the equipment. The degree of crystallinity of the PP in the films (X_c_) was determined using the following equation:

(1) X_c_ = ΔH_f_/ΔH_o_

where ΔH_f_ is the heat of fusion of the composite film and ΔH_ο_ is the heat of fusion for 100% crystalline PP (ΔH_100_ = 209 J/g) [2,19].

#### 3.3.5. Tensile Strength Analysis

The mechanical properties were measured using a universal testing machine Shimadzu AGS-X (Shimadzu Scientific Instruments, Columbia, MD, USA) equipped with a load cell of 100 N at a speed of 1 mm·min^−1^. The analysis was performed according to the ASTM D-1708-96 Norm. Ten specimens of each film were tested, and the average values are presented in this paper. The thickness of the film was measured using a Mitutoyo micrometer (Mitutoyo, Naucalpan de Juárez, Mexico).

## 4. Conclusions

Extruded composites of PP/chitosan with a maximum chitosan content of 9% (w/w) and 5% (w/w) of PPgMA were obtained. The FT-IR studies revealed the possible interaction between chitosan and PPgMA. The SEM results found that the addition of the compatibilizer and smaller particle size of the biopolymer resulted in the better distribution of the latter in the PP matrix, obtaining more homogeneous films. Moreover, the addition of chitosan decreased the contact angle of the films due to its high hydrophilicity. Finally, the crystallinity and the mechanical properties of PP decreased with the addition of chitosan, as expected due to the poor mechanical properties of this biopolymer. However, the addition of chitosan to PP provides the characteristics of natural polymers that increase the potential applications of this polymer, mainly in the production of friendly materials with the environment. In addition, due to the characteristic antimicrobial activity of chitosan, this material can be potentially applied in food containers to favor the preservation of food for a longer time in comparison with conventional materials.

## Figures and Tables

**Table 1:** Values of contact angles for PP film and PP/chitosan films.

Film	Contact Angle (°)
PP	104.7 ± 1.33
A5	82.7 ± 0.70
A7	79.8 ± 1.41
A9	78.3 ± 0.97
B5	78.8 ± 1.02
B7	78.9 ± 1.85
B9	78.4 ± 1.80
C5	81.0 ± 2.04
C7	76.8 ± 1.78
C9	74.5 ± 1.59

**Table 2:** Melting temperature (T_m_), fusion enthalpy (ΔH_f_), and crystallinity degree (X_c_) of films of PP and composites.

Film	T_m_ (°C)	ΔH_f_ (J/g)	X_c_ (%)
PP	165	96.69	46.26
PPgMA	157	77.16	36.92
A5	166	76.26	36.49
A7	165	61.07	29.22
A9	167	68.83	32.93
B5	166	70.13	33.55
B7	165	68.84	32.94
B9	167	66.89	32.01
C5	166	81.03	38.77
C9	166	66.48	31.81
C9	167	63.69	30.48

**Table 3:** Mechanical properties in PP/chitosan composites.

CODE	Tensile Strength (Mpa)	Young’s Modulus (Mpa)	Elongation at Break (%)
PP	41.55 ± 1.14	1484.00 ± 37.85	396.79 ± 12.91
A5	5.71 ± 0.44	282.33 ± 24.27	4.16 ± 0.23
A7	4.86 ± 0.43	272.64 ± 17.71	3.58 ± 0.29
A9	5.60 ± 0.68	306.44 ± 22.44	2.56 ± 0.26
B5	5.97 ± 0.74	505.44 ± 26.15	4.58 ± 0.11
B7	3.00 ± 0.10	266.82 ± 16.00	4.45 ± 0.82
B9	7.71 ± 0.34	384.44 ± 6.52	3.49 ± 0.15
C5	5.06 ± 0.33	350.70 ± 8.93	3.20 ± 0.20
C7	8.22 ± 0.40	363.71 ± 17.69	10.47 ± 1.66
C9	7.93 ± 0.43	435.28 ± 19.57	10.27 ± 0.48

**Table 4:** Concentration of each component used in the preparation of PP/chitosan composites.

	Composition of Films		
CODE	PP (wt %)	Chitosan (wt %)	PPgMA (wt %)
PP	100	0	0
A5	95	5	0
A7	93	7	0
A9	91	9	0
B5, C5 *	90	5	5
B7, C7 *	88	7	5
B9, C9 *	86	9	5

* films with milled chitosan. Note: the formulations contain 1 g glycerol/g chitosan.
